# Prevalence, sociodemographic determinants and regional disparities in health facility delivery in Somalia

**DOI:** 10.1007/s43999-026-00090-8

**Published:** 2026-03-25

**Authors:** Salad Halane, Abdiwali Ahmed, Mohamed Mustaf Ahmed, Jamilu Sani, Jamal Hassan Mohamoud

**Affiliations:** 1 Department of Public Health, Ministry of Health, Galmudug, Somalia; 2 Department of Health System Strengthening, Ministry of Health, Galmudug, Somalia; 3 Faculty of Medicine and Health Sciences, SIMAD University, Mogadishu, Somalia; 4 Department of Demography and Social Statistics, Federal University Birnin Kebbi, Birnin Kebbi, Kebbi State Nigeria; 5 Department of Public Health, Faculty of Medicine and Health Sciences, SIMAD University, Mogadishu, Somalia

**Keywords:** Health facility delivery, Maternal health, Sociodemographic determinants, Regional disparities, Somalia, SDHS, Skilled birth attendance, Healthcare access, Maternal mortality

## Abstract

**Background:**

Maternal mortality in Somalia remains alarmingly high, with an estimated ratio of 692 deaths per 100,000 live births. Health facility delivery, a key intervention for reducing maternal and neonatal mortality, is underutilized, with only 22.3% of women delivering to health facilities. This study examined the prevalence, regional disparities, and sociodemographic determinants of health facility delivery among Somali women.

**Methods:**

Data from 8,951 women aged 15–49 years who reported their place of delivery in the Somali Demographic and Health Survey (SDHS) were analyzed. Descriptive statistics were used to assess the prevalence of health facility deliveries and regional disparities. Bivariate and multivariable logistic regression models were used to identify sociodemographic factors associated with health facility delivery, including education, wealth, residence, and geographic region.

**Results:**

The overall prevalence of health facility deliveries was 22.3% with substantial regional variation. Woqooyi Galbeed had the highest prevalence (58%), while Bakool recorded the lowest (2%). Women with higher education (AOR: 4.24, 95% CI: 2.16–8.32) and those in the highest wealth quintile (AOR: 6.01, 95% CI: 4.04–8.94) were significantly more likely to deliver at health facilities. Rural and nomadic residences as well as distance to health facilities were associated with lower odds of health facility delivery.

**Conclusion:**

Education, wealth, and geographic access significantly influence health facility delivery in Somalia. Addressing these disparities through targeted investments in education, healthcare infrastructure, and culturally tailored interventions is essential to improve maternal health outcomes.

## Background

Maternal mortality remains a significant global health issue, with approximately 295,000 women dying annually from pregnancy-related complications, most of which are [1]. Sub-Saharan Africa bears a disproportionate burden, accounting for over 66% of these deaths [2]. Primary causes include hemorrhage, sepsis, hypertensive disorders, and obstructed labor, all of which can be effectively managed through timely access to skilled care during childbirth [3, 4]. Health facility delivery is a crucial intervention to reduce maternal mortality and improve neonatal outcomes by ensuring the presence of skilled birth attendants and access to emergency obstetric care [5, 6].

In Somalia, a country grappling with prolonged conflict and instability, the maternal mortality ratio (MMR) is alarmingly high, at 692 deaths per 100,000 live births, one of the highest globally [7–9]. Despite the importance of health facility delivery in mitigating these risks, usage remains critically low, with only 22.3% of women delivering to health facilities [10–12]. The majority of Somali women give birth at home without skilled assistance, significantly increasing the likelihood of adverse maternal and neonatal outcomes [1]. Addressing these gaps is vital for reducing preventable deaths and achieving global maternal health targets, including Sustainable Development Goal 3.1, which aims to lower the global MMR to fewer than 70 deaths per 100,000 live births by 2030 [13–15].

Sociodemographic factors such as education and economic status significantly influence health facility delivery. Higher educational levels enhance women’s health literacy, enabling them to recognize the benefits of skilled care during childbirth and navigate barriers to healthcare access [16, 17]. However, in Somalia, 83.7% of women lack formal education [18, 19]. Economic status also plays a crucial role: wealthier women are more capable of providing healthcare costs and transportation [20, 21], while approximately 69% of the population lives below the poverty line, making financial constraints a major barrier to accessing maternal healthcare [22, 23]. Geographic disparities complicate this scenario. Urban women are more likely to deliver in health facilities because of better access to healthcare infrastructure compared to their rural and nomadic counterparts, who face long travel distances and inadequate transportation options [24, 25]. Regional variations are stark; for instance, Woqooyi Galbeed has a health facility delivery rate of 58%, while Bakool reports a rate as low as 2% [6, 25]. These disparities reflect broader inequities in the healthcare infrastructure and socioeconomic development across Somalia.

Cultural and religious beliefs also affect delivery practice. In many Somali communities, home births are viewed as traditional or virtuous, leading to the perception that health facility deliveries are unnecessary or culturally inappropriate. Some religious beliefs may discourage seeking modern healthcare services [25, 26]. Addressing these cultural barriers is essential for improving maternal healthcare utilization. Evidence from other sub-Saharan African countries indicates that engaging community and religious leaders in culturally sensitive health promotion can significantly enhance health-seeking behaviors [27, 28].

Efforts to improve maternal healthcare in Somalia have encountered numerous challenges. Although international partners and NGOs have initiated programs to strengthen maternal health services, these efforts often suffer from limited funding, poor infrastructure, and insecurity in conflict-affected areas [27, 29]. Additionally, many programs do not adequately address the underlying sociodemographic and cultural determinants of healthcare utilization, which are crucial for sustainable improvements in maternal health outcomes [30]. This study aimed to examine the prevalence, sociodemographic determinants, and regional disparities in health facility delivery among Somali women, using data from the 2020 Somali Demographic and Health Survey (SDHS). By identifying factors such as education level, wealth status, residence type, and cultural influences, this study seeks to provide evidence-based insights that can inform targeted interventions. Addressing these barriers is critical for reducing maternal mortality rates and promoting equitable access to maternal healthcare in Somalia. This rewritten background maintains essential information while enhancing clarity and coherence.

## Methods

### Study design and data source

This study utilized a cross-sectional approach, leveraging data from the 2020 Somalia Demographic and Health Survey (SDHS). The SDHS, a nationally representative survey, provides extensive demographic and health-related information on women of reproductive age (15–49 years). In this study, the individual receiver (IR) dataset was analyzed, focusing on reproductive health outcomes among women.

### Study population and sampling

This study targeted women aged 15–49 years who participated in the 2020 SDHS. The analysis included women who provided information about their delivery location for their most recent childbirth, resulting in a sample size of 8,951 respondents.

### Ethical considerations

The SDHS adhered to ethical standards with approval from the National Health Research Ethics Committee of Somalia and the ICF Institutional Review Board. Participants provided informed consent before participating in the survey. This study involved secondary analysis of anonymized, publicly available data, eliminating the need for additional ethical approval.

### Study variables

The study’s main outcome variable was the place of delivery, categorized into two groups: health facility deliveries (coded as ‘1’) and home deliveries (coded as ‘0’). Health facility deliveries encompassed births in government hospitals, referral health units, mobile clinics, other public health facilities, private hospitals or clinics, and other private medical centers. Home deliveries referred to births at the respondent’s home or another home.

The independent variables included several sociodemographic characteristics such as age, educational attainment, marital status, religious affiliation, wealth index, distance to a health facility, employment status, number of living children, residence type (urban, rural, or nomadic), and regional location.

### Statistical analysis

Descriptive and inferential statistics were used to analyze the data. Descriptive statistics summarized the study population’s characteristics, while bivariate and multivariable logistic regression analyses assessed the associations between sociodemographic factors and delivery location. The analysis reported crude odds ratios (CORs) and adjusted odds ratios (AORs) with 95% confidence intervals (CIs), with statistical significance defined as *p* < 0.05.

Statistical analysis was conducted using Stata version 17, with sampling weights applied to account for complex survey design. Data visualization included pie charts and bar graphs, along with a spatial map generated using GeoPandas in Python, utilizing GADM map data for Somalia. These visualizations effectively highlight the prevalence of health facility deliveries, regional differences, and spatial distribution patterns across the country.

## Results

### Sociodemographic characteristics of women

The study included 8,951 women aged 15–49 years who reported their place of delivery for their most recent birth. Table 1 summarizes the respondents’ sociodemographic characteristics. The highest proportion of women fell within the 25–29 age group (27.37%), followed by the 20–24 age group (20.31%), while the 45–49 age group represented the smallest proportion (2.27%). The majority of the participants had no formal education (83.70%), while only 1.22% had attained higher education. Most respondents were married (90.86%), with divorced and widowed women accounting for 6.50% and 2.64% of the sample, respectively.

Economic disparities were evident, as 44.87% of the participants were in the lowest and second wealth quintiles, while only 16.38% were in the highest wealth quintile. Regarding access to healthcare, 63.94% of women reported that distance to a health facility posed a “big problem.” Additionally, 93.31% of the women were not currently employed, and only 6.69% were working at the time of the survey. In terms of residence, 35.29% lived in nomadic settings, 34.74% in urban areas, and 29.98% in rural areas.

**Table 1:** Sociodemographic characteristics of women

Variable	Weighted Frequency	Percent
**Age in 5-year groups**		
15–19	552	6.17%
20–24	1,817	20.31%
25–29	2,449	27.37%
30–34	1,791	20.02%
35–39	1,499	16.75%
40–44	635	7.10%
45–49	203	2.27%
**Education**		
No Education	7,492	83.70%
Primary	1,077	12.03%
Secondary	273	3.05%
Higher	109	1.22%
**Marital Status**		
Married	8,133	90.86%
Divorced	582	6.50%
Widowed	236	2.64%
**Wealth Quintile**		
Lowest	2,180	24.35%
Second	1,837	20.52%
Middle	1,676	18.73%
Fourth	1,792	20.02%
Highest	1,466	16.38%
**Distance to Health Facility**		
Big problem	5,721	63.94%
Not a big problem	3,226	36.06%
**Currently Working**		
Yes	599	6.69%
No	8,349	93.31%
**Number of Living Children**		
0–1	36	2.33%
2–3	302	19.51%
4–5	440	28.47%
6–7	457	29.55%
8+	312	20.14%
**Births in Last Five Years**		
0	366	4.09%
1	3,045	34.01%
2	3,422	38.23%
3	1,807	20.19%
4+	312	3.48%
**Residence**		
Urban	3,109	34.74%
Rural	2,683	29.98%
Nomadic	3,158	35.29%
**Region**		
Awdal	758	8.47%
Woqooyi Galbeed	536	5.99%
Togdheer	282	3.15%
Sool	701	7.83%
Sanaag	910	10.17%
Bari	557	6.23%
Nugaal	985	11.00%
Mudug	1,160	12.96%
Galgaduud	970	10.84%
Hiraan	597	6.67%
Middle Shabelle	334	3.74%
Banadir	482	5.39%
Bay	87	0.97%
Bakool	341	3.81%
Gedo	105	1.18%
Lower Juba	146	1.63%

### Prevalence and regional disparities in health facility delivery

**Fig. 1:**
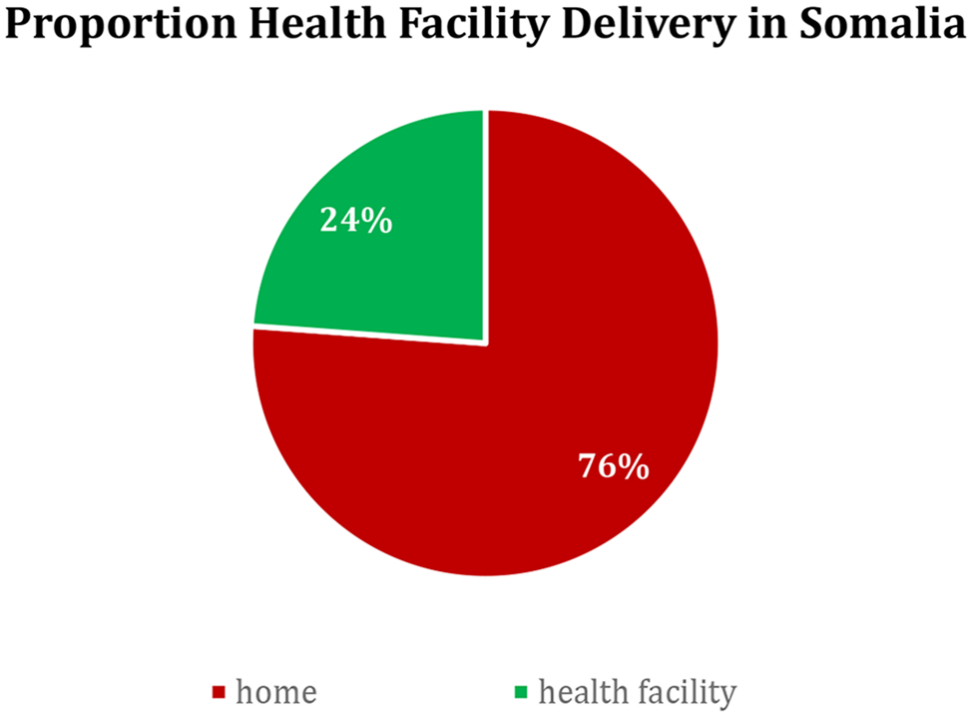
Prevalence of health facility delivery in Somalia

**Fig. 2:**
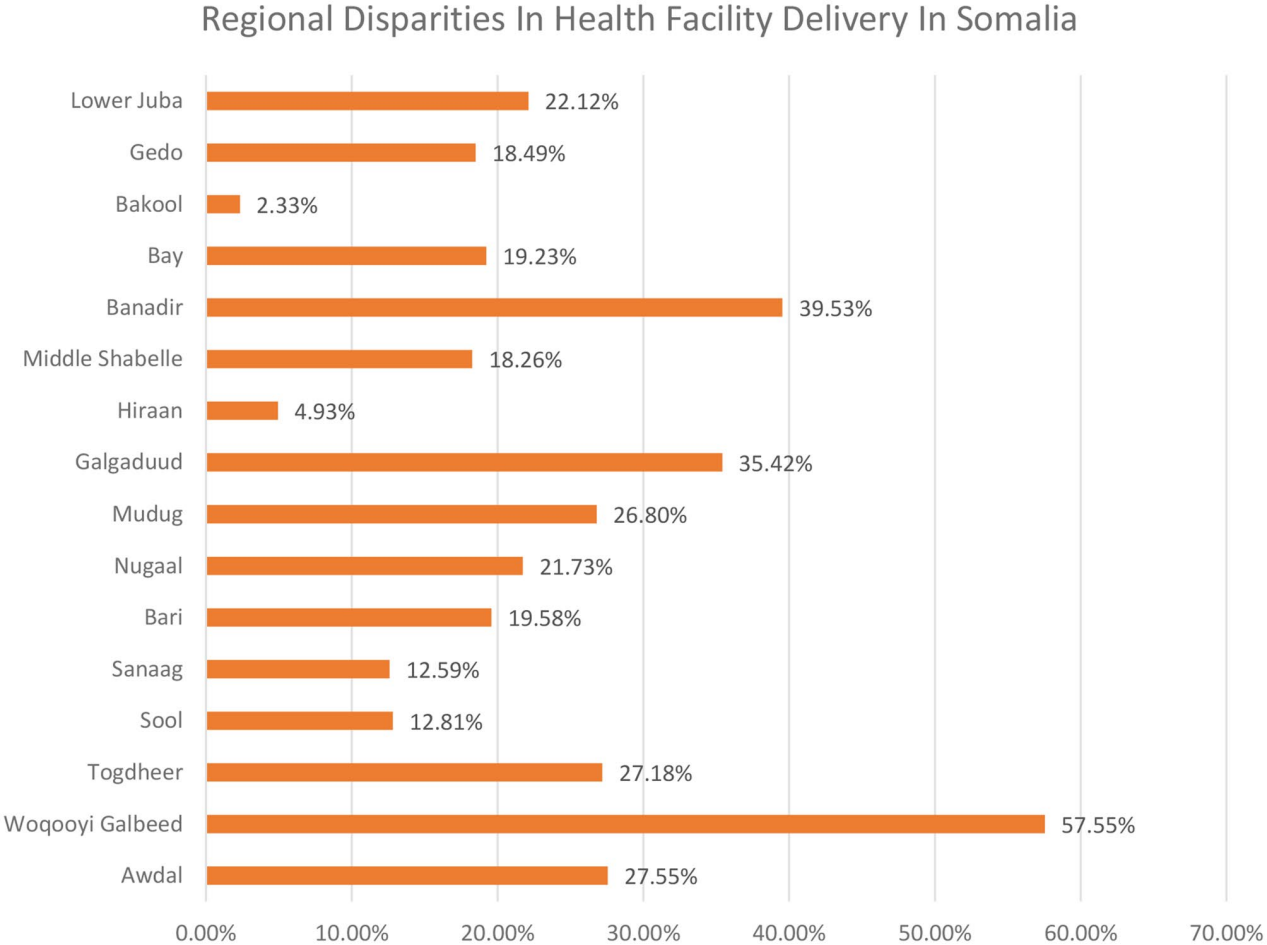
Regional disparities in health facility delivery across regions of Somalia

**Fig. 3:**
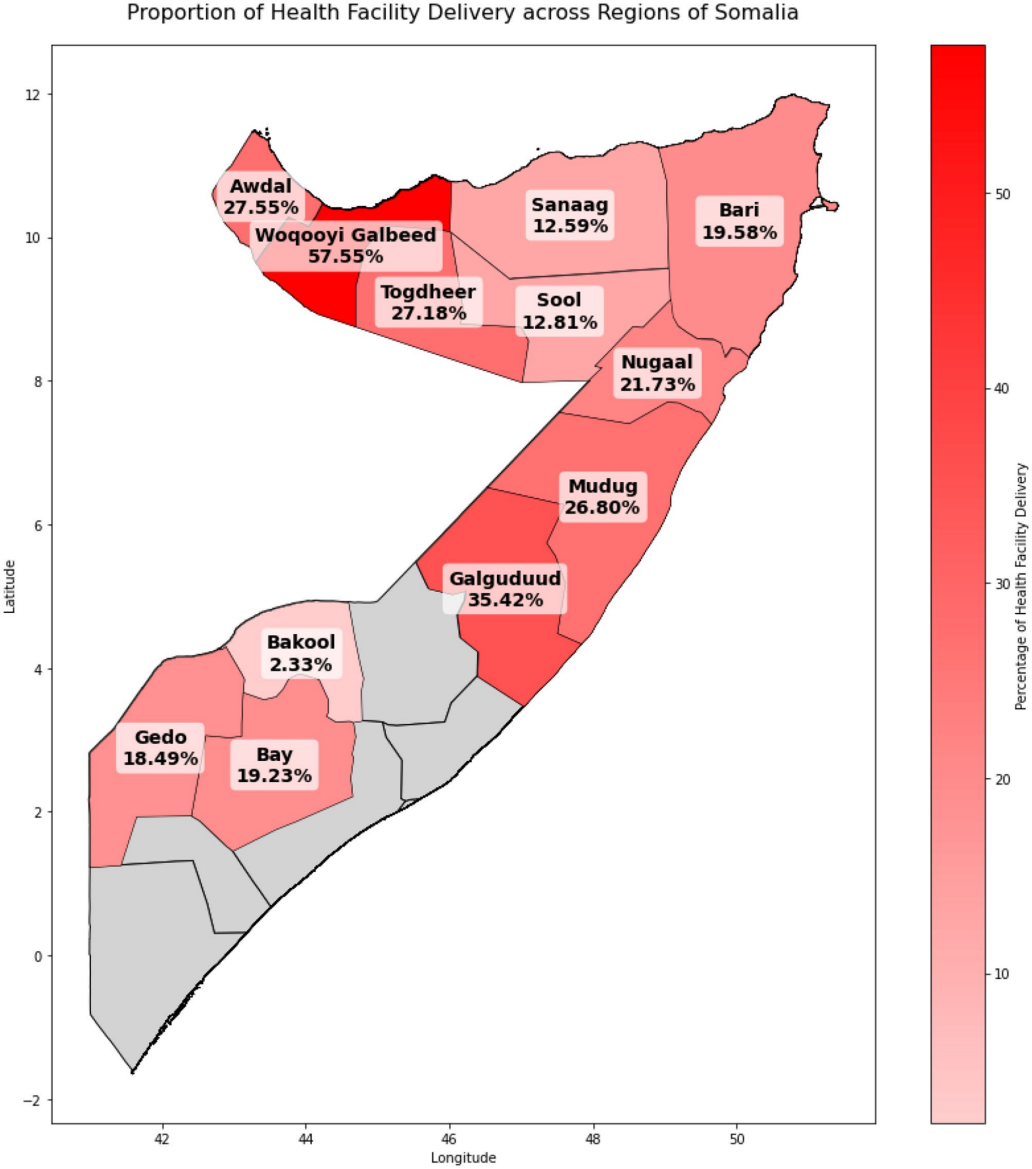
Map of Somalia showing proportion of health facility delivery across the regions

The overall prevalence of health facility deliveries in Somalia was 22.3%, as shown in Fig. 1. Regional variations were striking, with Woqooyi Galbeed recording the highest proportion of health facility deliveries (58%), and Bakool the lowest (2%). Regions such as Sanaag (13%) and Sool (13%) also had notably lower rates. These disparities are visually represented in Figs. 2 and 3, highlighting a clear geographic divide in health facility delivery rates across Somalia.

### Bivariate analysis of sociodemographic determinants of health facility delivery

Table 2 presents the results of the bivariate analysis. Education has emerged as a strong determinant of health facility delivery. Women with higher education were significantly more likely to deliver in a health facility (COR: 13.58, 95% CI: 7.32–25.19), followed by those with secondary education (COR: 7.40, 95% CI: 5.44–10.08) and primary education (COR: 3.72, 95% CI: 3.10–4.45), compared to women with no education.

Economic status also significantly influenced delivery locations. Women in the highest wealth quintile were far more likely to deliver in health facilities (COR: 18.95, 95% CI: 14.03–25.60) than those in the lowest quintile. Similarly, women who reported that distance to a health facility was “not a big problem” were more likely to deliver in a health facility (COR: 1.82, 95% CI: 1.60–2.09) compared to those who identified distance as a major barrier.

The type of residence had a notable impact on health facility delivery. Women in urban areas had a higher prevalence of health facility delivery (40%) than those in rural (27%) and nomadic areas (5%), with urban women showing significantly greater odds of facility-based deliveries (COR: 1.00, 95% CI: 0.48–0.65) than their rural and nomadic counterparts.

**Table 2:** Bivariate analysis of sociodemographic characteristics with health facility delivery

Variable	Place of Delivery		COR [95% C.I]	*P*-value
	Home Freq. (%)	Health Facility Freq. (%)		
**Age in 5-year groups**				
15–19 (Ref)	409 (74%)	142 (26%)	1	-
20–24	1,336 (74%)	480 (26%)	1.03[0.77–1.38]	0.833
25–29	1,839 (75%)	609 (25%)	0.95[0.72–1.26]	0.726
30–34	1,367 (76%)	423 (24%)	0.89[0.66–1.19]	0.434
35–39	1,170 (78%)	328 (22%)	0.81[0.60–1.09]	0.163
40–44	521 (82%)	114 (18%)	0.63[0.43–0.93]	0.019
45–49	168 (83%)	35 (17%)	0.60[0.34–1.05]	0.073
**Education**				
No Education (Ref)	6,105 (81%)	1,386 (19%)	1	-
Primary	584 (54%)	492 (46%)	3.72[3.10–4.45]	0.000
Secondary	101 (37%)	171 (63%)	7.40[5.44–10.08]	0.000
Higher	26 (24%)	82 (76%)	13.58[7.32–25.19]	0.000
**Marital Status**				
Married (Ref)	6,264 (77%)	1,867 (23%)	1	-
Divorced	379 (65%)	202 (35%)	1.78[1.41–2.26]	0.000
Widowed	172 (73%)	63 (27%)	1.24[0.84–1.81]	0.276
**Wealth Quintile**				
Lowest (Ref)	2,051 (94%)	128 (6%)	1	-
Second	1,656 (90%)	180 (10%)	1.74[1.22–2.47]	0.002
Middle	1,272 (76%)	404 (24%)	5.06[3.71–6.91]	0.000
Fourth	1,168 (65%)	623 (35%)	8.52[6.32–11.48]	0.000
Highest	669 (46%)	796 (54%)	18.95[14.03–25.60]	0.000
**Distance to Health Facility**				
Big problem (Ref)	4,588 (80%)	1,132 (20%)	1	-
Not a big problem	2,224 (69%)	1,001 (31%)	1.82[1.60–2.09]	0.000
**Currently Working**				
Yes (Ref)	410 (69%)	188 (31%)	1	-
No	6,404 (77%)	1,944 (23%)	0.66[0.52–0.84]	0.001
**Number of Living Children**				
0–1 (Ref)	22 (63%)	13 (37%)	1	-
2–3	222 (74%)	79 (26%)	0.61[0.23–1.57]	0.304
4–5	348 (79%)	92 (21%)	0.45[0.18–1.16]	0.099
6–7	370 (81%)	86 (19%)	0.40[0.16–1.02]	0.055
8 +	244 (79%)	67 (21%)	0.47[0.18–1.21]	
**Births in Last Five Years**				
0 (Ref)	289 (79%)	76 (21%)	1	-
1	2,266 (74%)	778 (26%)	1.31[0.92–1.87]	0.135
2	2,631 (77%)	790 (23%)	1.15[0.81–1.63]	0.448
3	1,382 (76%)	424 (23%)	1.17[0.81–1.69]	0.404
4+	246 (79%)	65 (21%)	1.01[0.62–1.64]	0.956
**Residence**				
Urban (Ref)	1,868 (60%)	1,240 (40%)	1	-
Rural	1,959 (73%)	723 (27%)	0.56[0.48–0.65]	0.000
Nomadic	2,989 (95%)	169 (5%)	0.09[0.07–0.11]	0.000
**Region**				
Woqooyi Galbeed	549 (72%)	208 (28%)	1	-
Awdal	227 (42%)	308 (58%)	0.28 [0.19–0.40]	0.000
Togdheer	205 (73%)	76 (27%)	0.28 [0.21–0.36]	0.000
Sool	610 (87%)	90 (13%)	0.11 [0.08–0.15]	0.000
Sanaag	795 (87%)	114 (13%)	0.11 [0.08–0.14]	0.000
Bari	448 (80%)	109 (20%)	0.18 [0.13–0.25]	0.000
Nugaal	770 (78%)	213 (22%)	0.20 [0.15–0.28]	0.000
Mudug	849 (73%)	311 (27%)	0.27 [0.20–0.36]	0.000
Galgaduud	626 (65%)	343 (35%)	0.40 [0.30–0.54]	0.000
Hiraan	567 (95%)	29 (5%)	0.04 [0.02–0.06]	0.000
Middle Shabelle	273 (82%)	61 (18%)	0.16 [0.11–0.24]	0.000
Banadir	291 (60%)	190 (40%)	0.48 [0.38–0.61]	0.000
Bay	69 (81%)	16 (19%)	0.18 [0.12–0.26]	0.000
Bakool	332 (98%)	8 (2%)	0.02 [0.01–0.03]	0.000
Gedo	85 (81%)	19 (19%)	0.17 [0.12–0.24]	0.000
Lower Juba	113 (78%)	32 (22%)	0.21 [0.15–0.30]	0.000

### Multivariable analysis of factors associated with health facility delivery

Multivariate analysis confirmed that education, wealth status, and residence were the strongest predictors of health facility delivery (Table 3). Women with higher education had over four times the odds of delivering in a health facility (AOR: 4.24, 95% CI: 2.16–8.32), whereas those with secondary education were nearly three times as likely (AOR: 2.80, 95% CI: 1.99–3.95).

Economic disparities persist in the adjusted model. Women in the highest wealth quintile were six times more likely to deliver at a health facility (AOR: 6.01, 95% CI: 4.04–8.94) than those in the lowest quintile. Similarly, urban residence was associated with significantly higher odds of health facility delivery (AOR: 1.00) than rural (AOR: 0.71, 95% CI: 0.59–0.86) and nomadic settings (AOR: 0.23, 95% CI: 0.16–0.32).

Regionally, Woqooyi Galbeed remained the reference category because of its relatively high prevalence in health facility delivery (58%). Regions such as Bakool (AOR, 0.04; 95% CI, 0.02–0.06) and Hiraan (AOR, 0.04; 95% CI, 0.02–0.06) had the lowest odds of health facility delivery.

**Table 3:** Multivariable analysis of sociodemographic determinants of health facility delivery

Category	Place of Delivery		AOR [95% C.I]	*P*-value
	Home Freq. (%)	Health Facility Freq. (%)		
**Age in 5-year groups**				
15–19	409 (74%)	142 (26%)	ref.	
20–24	1,336 (74%)	480 (26%)	0.87 [0.62–1.22]	0.427
25–29	1,839 (75%)	609 (25%)	0.79 [0.57–1.10]	0.16
30–34	1,367 (76%)	423 (24%)	0.72 [0.51–1.02]	0.064
35–39	1,170 (78%)	328 (22%)	0.69 [0.48–0.98]	0.038
40–44	521 (82%)	114 (18%)	0.58 [0.37–0.90]	0.016
45–49	168 (83%)	35 (17%)	0.49 [0.25–0.98]	0.045
**education_level**				
No education	6,105 (81%)	1,386 (19%)	ref.	
Primary	584 (54%)	492 (46%)	1.92 [1.55–2.38]	0.000
Secondary	101 (37%)	171 (63%)	2.80 [1.99–3.95]	0.000
Higher	26 (24%)	82 (76%)	4.24 [2.16–8.32]	0.000
**marital status**				
married	6,264 (77%)	1,867 (23%)	ref.	
Divorced	379 (65%)	202 (35%)	1.11 [0.84–1.47]	0.474
Widowed	172 (73%)	63 (27%)	1.45 [0.94–2.25]	0.096
**wealth_quintile**				
lowest	2,051 (94%)	128 (6%)	ref.	
Second	1,656 (90%)	180 (10%)	1.71 [1.15–2.56]	0.008
Middle	1,272 (76%)	404 (24%)	2.36 [1.58–3.53]	0.000
Fourth	1,168 (65%)	623 (35%)	3.60 [2.42–5.37]	0.000
Highest	669 (46%)	796 (54%)	6.01 [4.04–8.94]	0.000
**distance to health facility**				
big problem	4,588 (80%)	1,132 (20%)	ref.	
Not a big problem	2,224 (69%)	1,001 (31%)	1.09 [0.93–1.28]	0.283
**work status**				
yes	410 (69%)	188 (31%)	ref.	
No	6,404 (77%)	1,944 (23%)	0.99 [0.74–1.33]	0.946
**Residence**				
urban	1,868 (60%)	1,240 (40%)	ref.	
Rural	1,959 (73%)	723 (27%)	0.71 [0.59–0.86]	0.000
Nomadic	2,989 (95%)	169 (5%)	0.23 [0.16–0.32]	0.000
**Region**				
Woqooyi Galbeed	227 (42%)	308 (58%)	ref.	-
Awdal	549 (72%)	208 (28%)	0.80 [0.54–1.20]	0.286
Togdheer	205 (73%)	76 (27%)	0.37 [0.27–0.50]	0.000
Sool	610 (87%)	90 (13%)	0.18 [0.13–0.26]	0.000
Sanaag	795 (87%)	114 (13%)	0.10 [0.07–0.14]	0.000
Bari	448 (80%)	109 (20%)	0.18 [0.12–0.27]	0.000
Nugaal	770 (78%)	213 (22%)	0.19 [0.14–0.28]	0.000
Mudug	849 (73%)	311 (27%)	0.23 [0.16–0.31]	0.000
Galgaduud	626 (65%)	343 (35%)	0.45 [0.32–0.63]	0.000
Hiraan	567 (95%)	29 (5%)	0.04 [0.02–0.06]	0.000
Middle Shabelle	273 (82%)	61 (18%)	0.18 [0.12–0.26]	0.000
Banadir	291 (60%)	190 (40%)	0.20 [0.15–0.27]	0.000
Bay	69 (81%)	16 (19%)	0.11 [0.07–0.17]	0.000
Bakool	332 (98%)	8 (2%)	0.04 [0.02–0.06]	0.000
Gedo	85 (81%)	19 (19%)	0.22 [0.15–0.32]	0.000
Lower Juba	113 (78%)	32 (22%)	0.13 [0.09–0.20]	0.000

## Discussion

This study highlights the alarmingly low prevalence of health facility delivery in Somalia (22.3%) with stark sociodemographic and regional disparities. These findings align with previous research from sub-Saharan Africa, which documents similarly low utilization of health facilities for childbirth in resource-poor settings [25, 31]. These results emphasize the urgent need for targeted interventions to address barriers to health facility delivery, particularly in rural and underserved regions. Education has emerged as a critical determinant of health facility delivery. Women with secondary education were nearly three times more likely to deliver in health facilities (AOR: 2.80, 95% CI: 1.99–3.95), while those with higher education had over four times the odds ratio (AOR: 4.24, 95% CI: 2.16–8.32) compared to women with no formal education. Education equips women with health literacy and decision-making autonomy, enabling them to recognize the benefits of skilled birth attendance and effectively navigate healthcare systems effectively [16, 32]. In Somalia, where 83.7% of women lack formal education, addressing educational inequities is essential for improving maternal healthcare utilization. Initiatives such as female education programs and community-based health literacy campaigns could significantly enhance awareness and acceptance of health facility delivery [16]. Evidence from other LMICs suggests that increased education leads to long-term generational improvements in maternal and child health outcomes [16]. Economic disparities also strongly influence the delivery of health facilities. Women in the highest wealth quintile were six times more likely to deliver in health facilities (AOR: 6.01, 95% CI: 4.04–8.94) compared to those in the poorest quintile. Financial constraints remain a significant barrier to maternal healthcare access in Somalia, where nearly 70% of the population live below the poverty line [33, 34].

Wealthier women are more likely to afford transportation, healthcare costs, and associated expenses, while poorer women face competing priorities that discourage health-seeking behaviors [35]. Addressing this disparity through financial assistance programs such as conditional cash transfers or subsidized healthcare services could reduce the economic burden and encourage greater utilization of health facilities. Studies in similar contexts such as Ethiopia and Nigeria have demonstrated that such interventions are effective in increasing facility-based deliveries [36–39]. Geographic disparities were pronounced, with Woqooyi Galbeed reporting the highest prevalence of health facility delivery (58%), and Bakool the lowest (2%). Urban women had significantly higher odds of facility-based deliveries (40%) than rural (27%) and nomadic (5%) women. These findings highlight the role of healthcare infrastructure and accessibility in influencing delivery locations. Urban areas tend to have better proximity to healthcare facilities, transportation networks, and skilled birth attendants, while rural and nomadic populations face logistical challenges such as long travel distances, poor roads, and limited availability of healthcare services [24, 33, 40]. Women who reported that distance was “not a big problem” had significantly higher odds of delivering in a health facility (AOR: 1.82, 95% CI: 1.60–2.09), underscoring the importance of physical accessibility in maternal healthcare utilization. Investments in rural healthcare infrastructure, mobile health units, and transportation support are critical for bridging these gaps.

Cultural and religious norms also play a substantial role in shaping delivery practice. In many Somali communities, home births are perceived as traditional or virtuous, whereas health facility deliveries are often viewed as unnecessary or culturally inappropriate [41]. Religious beliefs may further discourage the use of modern healthcare, particularly in conservative communities [28]. Addressing these barriers requires culturally sensitive interventions that engage community and religious leaders to promote health facility delivery as a safe and acceptable practice. Evidence from other sub-Saharan African countries demonstrates that culturally tailored health education campaigns can significantly improve maternal health-seeking behaviors [42, 43]. Age was another factor that influenced health facility deliveries. Women aged 35–39 years were more likely to deliver in health facilities (47.16%) than younger women aged 15–19 years (28.03%). Younger women may lack decision-making autonomy or be less aware of the benefits of skilled care, reflecting broader gender and age-related inequalities [35]. Programs targeting younger mothers, particularly adolescents, should focus on empowering them with information and support to access maternal healthcare services [44].

### Limitations

This study has several limitations that should be acknowledged. The cross-sectional design restricts the ability to establish causal relationships between sociodemographic factors and health facility delivery, as data were captured at a single point in time. Additionally, reliance on self-reported data from the Somali Demographic and Health Survey (SDHS) may introduce recall and reporting biases, potentially compromising result accuracy. Geographic representation may also be limited, as populations in remote or conflict-affected areas, including nomadic groups, are likely underrepresented, which could lead to an underestimation of disparities in health facility delivery. Furthermore, important unmeasured confounders such as cultural beliefs, decision-making autonomy, and specific characteristics of healthcare facilities were not included, which could significantly influence maternal healthcare utilization. The data were collected in 2020, meaning recent changes in healthcare infrastructure or sociocultural dynamics may not be reflected in the findings. Finally, the results are specific to Somalia and may not be generalizable to other low-resource or conflict-affected settings. These limitations highlight the need for further research to address these gaps and validate the findings across different contexts.

## Conclusion

This study highlights the critically low prevalence of health facility delivery in Somalia, with only 22.3% of women utilizing these services. Significant disparities were identified across regions, socioeconomic groups, and geographical settings. Women with higher education and those in the wealthiest quintile were significantly more likely to deliver in health facilities, underscoring the importance of education and economic stability in improving maternal healthcare utilization. In contrast, rural and nomadic populations, particularly in regions such as Bakool and Sanaag, experienced the lowest rates of facility-based deliveries due to infrastructural deficiencies and cultural barriers. Addressing these disparities is essential for reducing maternal mortality and advancing equitable access to maternal healthcare.

Therefore, targeted interventions are necessary to overcome these challenges. Expanding educational opportunities for women and girls in underserved areas is critical for enhancing health literacy and empowering maternal health decisions. Investments in rural healthcare infrastructure, including the establishment of mobile health units, can bridge geographic gaps and improve accessibility. Financial support mechanisms such as subsidies for maternal healthcare or transportation vouchers are vital for mitigating economic barriers. In addition, culturally sensitive awareness campaigns that engage communities and religious leaders can address resistance rooted in traditional norms and beliefs. Finally, healthcare policies should focus on region-specific strategies, prioritizing underserved areas, such as Bakool and Sanaag, to ensure equitable access. These efforts, coupled with sustained investments and community engagement, can significantly reduce maternal mortality and help Somalia achieve global health targets, such as Sustainable Development Goal 3.1.

## Data Availability

The data that support the findings of this study are available in the 2020 Somali Demographic and Health Survey (SDHS) at https://nbs.gov.so/wp-content/uploads/2023/07/Somali-Health-Demographic-Survey-2020.pdf. The data were derived from the following resources available in the public domain:
